# Clinical and molecular epidemiological features of critically ill patients with invasive group A *Streptococcus* infections: a Belgian multicenter case-series

**DOI:** 10.1186/s13613-024-01249-7

**Published:** 2024-01-29

**Authors:** Marijke Peetermans, Veerle Matheeussen, Cedric Moerman, Fréderic De Rydt, Sabine Thieren, Emily Pollet, Michael Casaer, Benjamin De Backer, Rudi De Paep, Yves Debaveye, Lars Desmet, Stefanie Desmet, Els I. M. Duval, Vincent Fraipont, Dieter Geysels, Greet Hermans, Frederik Lahaye, Xavier Mathy, Philippe Meersseman, Cécile Meex, Jozef Van Herck, Stefanie van Kleef-van Koeveringe, Nathalie Layios, Joost Wauters, Philippe G. Jorens

**Affiliations:** 1 Medical Intensive Care Unit, Department of General Internal Medicine, University Hospitals Leuven, Herestraat 49, 3000 Leuven, Belgium; 2 Laboratory for Clinical Infectious and Inflammatory Disorders, Department of Microbiology, Immunology and Transplantation, KU Leuven, Herestraat 49, 3000 Leuven, Belgium; 3 Department of Microbiology and Belgian Reference Centre for Invasive β-Hemolytic Streptococci, Antwerp University Hospital, Drie Eikenstraat 655, 2650 Edegem, Antwerp Belgium; 4 Laboratory of Medical Biochemistry and Laboratory of Medical Microbiology, University of Antwerp, Universiteitsplein 1, 2610 Wilrijk, Antwerp Belgium; 5 Department of Adult and Pediatric Intensive Care Medicine, Antwerp University Hospital, Drie Eikenstraat 655, 2650 Edegem, Antwerp Belgium; 6 Department of Intensive Care Medicine, University Hospitals Leuven, Herestraat 49, 3000 Leuven, Belgium; 7 Laboratory of Intensive Care Medicine, Department of Cellular and Molecular Medicine, KU Leuven, Herestraat 49, 3000 Leuven, Belgium; 8 Service de Microbiologie Clinique, CHR Citadelle, Bd du Douzième de Ligne 1, 4000 Liège, Belgium; 9 Pediatric Intensive Care Unit, Department of Intensive Care Medicine, University Hospitals Leuven, Herestraat 49, 3000 Leuven, Belgium; 10 Laboratory for Clinical Microbiology, Department of Microbiology, Immunology and Transplantation, KU Leuven, Herestraat 49, 3000 Leuven, Belgium; 11 Service des Soins Intensifs, CHR Citadelle, Bd du Douzième de Ligne 1, 4000 Liège, Belgium; 12 Service de Microbiologie Clinique, University Hospital Liège, Avenue de l’Hôpital, 4000 Liège, Belgium; 13 Department of Intensive Care, University Hospital Liège, Avenue de l’Hôpital, 4000 Liège, Belgium; 14 Département des Sciences Cliniques, University of Liège, 4000 Liège, Belgium; 15 Department of Medicine and Health Sciences, Laboratory of Experimental Medicine and Pediatrics (LEMP), University of Antwerp, Universiteitsplein 1, 2610 Wilrijk, Antwerp Belgium; 16 Present Address: Department of Anesthesiology, Chirec Hospitals, Brussels, Belgium; 17 Present Address: Department of Anesthesiology and Critical Care Medicine, GZA Hospital Group, Antwerp, Belgium; 18 Present Address: Department of Anesthesiology, VITAZ Hospital, Sint-Niklaas, Belgium

**Keywords:** Group A streptococci, Invasive, *Streptococcus pyogenes*, Necrotizing fasciitis, Toxic shock syndrome, Community-acquired pneumonia, Empyema, Viral coinfection, Influenza, Critical care

## Abstract

**Background:**

Recent alerts have highlighted an increase in group A streptococcal (GAS) infections since 2022 in Europe and the United States. *Streptococcus pyogenes* can cause limited skin or mucosal disease, but can also present as severe invasive disease necessitating critical care. We performed a multicenter retrospective study of patients with GAS infections recently admitted to Belgian intensive care units (ICUs) since January 2022. We describe patient characteristics and investigate the molecular epidemiology of the *S. pyogenes* strains involved.

**Results:**

Between January 2022 and May 2023, a total of 86 cases (56 adults, 30 children) with GAS disease were admitted to critical care in the university hospitals of Leuven, Antwerp and Liège. We noted a strikingly high incidence of severe community-acquired pneumonia (sCAP) (45% of adults, 77% of children) complicated with empyema in 45% and 83% of adult and pediatric cases, respectively. Two-thirds of patients with *S. pyogenes* pneumonia had viral co-infection, with influenza (13 adults, 5 children) predominating. Other disease presentations included necrotizing fasciitis (23% of adults), other severe skin/soft tissue infections (16% of adults, 13% of children) and ear/nose/throat infections (13% of adults, 13% of children). Cardiogenic shock was frequent (36% of adults, 20% of children). Fifty-six patients (65%) had toxic shock syndrome. Organ support requirements were high and included invasive mechanical ventilation (77% of adults, 50% of children), renal replacement therapy (29% of adults, 3% of children) and extracorporeal membrane oxygenation (20% of adults, 7% of children). Mortality was 21% in adults and 3% in children. Genomic analysis of *S. pyogenes* strains from 55 out of 86 patients showed a predominance of *emm1* strains (73%), with a replacement of the M1_global_ lineage by the toxigenic M1_UK_ lineage (83% of *emm1* strains were M1_UK_).

**Conclusions:**

The recent rise of severe GAS infections (2022–23) is associated with introduction of the M1_UK_ lineage in Belgium, but other factors may be at play—including intense circulation of respiratory viruses and potentially an immune debt after the COVID pandemic. Importantly, critical care physicians should include *S. pyogenes* as causative pathogen in the differential diagnosis of sCAP.

**Supplementary Information:**

The online version contains supplementary material available at 10.1186/s13613-024-01249-7.

## Background

*Streptococcus pyogenes (S. pyogenes)* can cause infections of varying severity, ranging from benign skin infections or scarlet fever to severe invasive infections such as necrotizing fasciitis and toxic shock syndrome (StrepTSS) [1]. Risk factors for invasive group A streptococcal (iGAS) infection include extremes of age, diabetes, alcohol and intravenous drug use, but up to a third of cases occur in patients without comorbidities [2]. Also, circulation of specific virulent *emm* types in the community may impact severity and clinical presentation of iGAS infections [3, 4]. Moreover, varicella zoster and influenza A viral infections have been associated with iGAS infections [2, 5, 6]. Recent data from the Brussels Capital region (Belgium) showed an increasing incidence of iGAS infections already before the COVID pandemic, from 2.1 to 10.9/100000 inhabitants [7]. However, this rising trend has not been uniformly observed in other countries [8, 9].

Several critical care units in Belgium noted an increased burden of severe iGAS infections mainly since autumn of 2022 in parallel with a doubling (+ 122%) in mandatory notifications of the disease in the three national surveillance networks [10]. In fact, recent alerts from the CDC and WHO [11, 12] highlighted outbreaks of pediatric scarlet fever and iGAS infections, but data from critical care have been lacking. Also, the clinical phenotypes of critical iGAS infections as seen during recent months, have not been described.

We designed a multicenter, retrospective cohort study in the Belgian university hospitals of Antwerp, Liège and Leuven, to describe the patient population, clinical presentation and outcomes of this new epidemic of iGAS disease necessitating critical care. We also performed genetic subtyping of the iGAS strains involved to ascertain whether circulation of a highly virulent subtype could explain the rise in critical iGAS cases.

## Methods

We retrospectively collected clinical data from patients with microbiologically documented severe infection with *S. pyogenes* who were consecutively admitted to the intensive care unit in the University Hospitals of Antwerp (UZA), Leuven (UZ Leuven) and Liège (CHU Liège and CHR Citadelle) between January 2022 and May 2023. Our hospitals are all tertiary referral centers that together cover a population of > 4 million inhabitants and have 211 ICU beds and 3385 hospital beds. Patients were considered to have definite iGAS infection if there was isolation of GAS from a normally sterile site (i.e., blood cultures, cerebrospinal, pleural or peritoneal fluid) as per the 1995 CDC case definition [13]; and probable iGAS infection if GAS was isolated from a non-sterile site (e.g., wound, genital, or respiratory tract) with clinical evidence of invasive infection such as necrotizing fasciitis, sepsis or toxic shock syndrome. Toxic shock syndrome was defined according to the 2010 CDC case definition [14] and requires the presence of hypotension with at least two of: renal failure, diffuse intravascular coagulation (DIC) or thrombocytopenia, liver involvement, acute respiratory distress syndrome, rash, soft tissue necrosis.

Data were collected from the patient electronic health records and entered into an anonymized database. We documented demographics, comorbidities, viral coinfections, clinical phenotype, laboratory characteristics and scores (ISTH score for DIC [15], APACHE II [16] and SOFA [17] as scores for severity of illness and organ failure, respectively), need for respiratory, cardiovascular or renal support, antibiotic therapy, use of intravenous immunoglobulins, length of stay and mortality. The Ethics committees of the participating hospitals waived the requirement for informed consent for this retrospective data collection.

*Streptococcus pyogenes* isolates from invasive infections are routinely sent, although not mandatory, to the Belgian Reference Laboratory at the Antwerp University Hospital for *emm* gene typing of all isolates (according to the CDC *emm* typing protocol [18]) as GAS strains are classified by genetic differences in the surface M protein encoded by the *emm* gene [19]. Whole genome sequencing (Illumina MiSeq) is performed on a selection of isolates in search of pathogen-specific factors such as superantigens and hypervirulent clones, like the toxigenic M1_UK_ variant [20, 21].

Data are reported as mean ± standard deviation, median (interquartile range), numbers or proportions, as appropriate. For comparison between groups, Mann–Whitney U and Chi square tests were used. We used Prism 9 (version 9.5.1, GraphPad Software) for these analyses. Adult and pediatric patient data are presented separately.

## Results

### Patient characteristics

During the study period, a total of 86 critically ill patients (of which 56 adults) with iGAS infection were identified. Three quarters of patients were referred from other hospitals, after a median of 1 day (IQR 0–3). Clinical characteristics of patients can be found in Table 1.

**Table 1:** Clinical data from 86 critically ill patients with invasive group A streptococcal infections

	Adult cases (*n* = 56)	Pediatric cases (*n* = 30)
Demographics		
Age (years)	48 ± 16	2 (1–5)
Male gender (*n*, %)	34 (61%)	17 (57%)
Weight (kg)	80 ± 19	14 (10–19)
BMI (kg/m^2^)	26 (23–28)	NA
Clinical presentation		
Duration of symptoms prior to hospital admission (days)	2 (1–4)	3 (2–5)
Pneumonia (*n*, %)	25 (45%)	23 (77%)
With empyema (*n*)	12	19
Necrotizing fasciitis (*n*, %)	13 (23%)	0
Other SSTI (*n*, %)	9 (16%)	4 (13%)
ENT infection (*n*, %)	7 (13%)	4 (13%)
Puerperal sepsis (*n*, %)	2 (4%)	NA
Toxic shock syndrome (*n*, %)	43 (77%)	13 (43%)
Viral co-infection (*n*, %)	18 (32%)	21 (70%)
Influenza	14	7
HMPV	3	4
RSV	1	3
SARS CoV-2	0	3
Varicella	0	3
Severity of illness and organ support		
APACHE II score	22 ± 9	21 ± 9
SOFA score at ICU admission	12 ± 5	NA
Highest SOFA score in ICU	13 ± 5	NA
Invasive mechanical ventilation at ICU admission	35 (63%)	9 (30%)
Invasive mechanic ventilation (*n*, %)	43 (77%)	15 (50%)
Ventilator days	12 (3–24)	4 (2–12)
RRT at ICU admission	13 (23%)	0
RRT (*n*, %)	16 (29%)	1 (3%)
Days on RRT	16 ± 13	15
Requiring vasoactive drugs at ICU admission	45 (80%)	9 (30%)
Septic shock (*n*, %)	49 (88%)	9 (30%)
Cardiogenic shock (*n*, %)	20 (36%)	6 (20%)
ECMO (*n*, %)	11 (20%)	2 (7%)
Days on ECMO	15 (10–18)	9 (8–10)
Outcomes		
ICU length-of-stay (days)	16 (6–40)	7 (4–17)
Hospital length-of-stay (days)	29 (20–65)	18 (16–30)
28 day mortality (*n*, %)	11 (20%)	1 (3%)
ICU mortality (*n*, %)	12 (21%)	1 (3%)
In-hospital mortality (*n*, %)	12 (21%)	1 (3%)

BMI: body mass index; *n* number of patients; NA: not applicable; ENT: ear, nose and throat; SSTI: skin or soft tissue infection; HMPV: human metapneumovirus; RSV: Respiratory syncytial virus; SARS-CoV-2: severe acute respiratory syndrome coronavirus 2; APACHE II: acute physiology, age and chronic health evaluation; SOFA: sequential organ failure assessment; RRT: renal replacement therapy; ECMO: extracorporeal membrane oxygenation; ICU: intensive care unit

Chronic airway disease (*n* = 8 patients), malignancies (*n* = 7), arterial hypertension (*n* = 7), immune suppression (*n* = 5), psychiatric disorders (*n* = 6), alcoholism (*n* = 5) and intravenous drug use (*n* = 4) were the most frequent comorbidities. Adults were often overweight or obese (median BMI of 26 kg/m^2^). About half of patients (21/56 adults and 24/30 children) had no underlying medical conditions. A large proportion of cases (45% of adults, 77% of children) presented with severe community-acquired pneumonia (sCAP) and many (77% of adults, 43% of children) had toxic shock syndrome. Indeed, lab results on admission to ICU showed thrombocytopenia (< 100*10^9^/L) in 40% of cases (34/86), overt DIC in 23% (20/86), acute liver injury in 34% (29/86) and acute kidney injury in 44% (38/86). In 37 adults and 15 children, *S. pyogenes* was cultured from blood cultures. Viral coinfection was documented in one out of three adults, and two out of three children, with influenza and HMPV predominating.

For pneumonia specifically, 16/25 adults and 17/23 children had evidence of viral coinfection. Conversely, of all patients with combined viral and severe GAS infection, the clinical presentation was pneumonia in 16/18 adults and 17/21 children. For pneumonia cases, the majority was multilobar (31/48 or 65%) and many were complicated with empyema. Eight patients with pneumonia even developed lung parenchymal necrosis and/or cavitation. The median pO_2_/F_i_O_2_ ratio at ICU admission was 140 (IQR 88–250). The highest number of ICU admissions occurred during winter, coinciding with the peak of the influenza epidemic in Belgium [22] (Additional file 1: Fig. S1).

Patients were severely ill, more than half required invasive ventilation, a third had cardiogenic shock and one in five required kidney support. Eleven adults and 2 children required extracorporeal membrane oxygenation, including 4 who required veno-arterial or veno-arteriovenous ECMO.

Patients were treated with appropriate antibiotics, including adjunctive clindamycin (in 50/56 adults and 25/30 children). Beta-lactam antibiotics for *S. pyogenes* infections were given for median 15 days (IQR 11–22) as some patients received prolonged courses for presentations such as empyema, arthritis, spondylodiscitis, mastoiditis, infective endocarditis. Clindamycin was given for 6 (IQR 4–8) days. Only 1 adult and 1 child were receiving antibiotics prior to hospital presentation. In three quarters of referred patients (48 of 64 referred cases), antibiotic therapy was started in the first hospital. For necrotizing fasciitis cases, surgery was performed either the day of admission, or the day thereafter, patients required a median of 4 (1.5–5) surgeries. Intravenous immunoglobulins were used less frequently in the total population (in 24/56 adults and 6/30 children) and in 27/56 patients with toxic shock syndrome.

One in five adults with critical iGAS infection did not survive their admission. In-hospital mortality from critical GAS pneumonia was 16% (4/25) in adults and 4% (1/23) in children; for necrotizing fasciitis case fatality rate was higher at 38% (5/13). Toxic shock syndrome was associated with a mortality of 26% (11/43) in adults and 8% (1/13) in children. Seventy-seven percent (10/13) of all patients who temporarily required ECMO survived. Co-isolation of viruses was not associated with a higher mortality rate (4/39 patients with viral co-infection, as compared to 9/47 patients without evidence for viral coinfection, died in hospital). Factors significantly associated with mortality in univariate analysis for the adult patients were severity of illness and organ failure (APACHE II score, SOFA score and receipt of invasive mechanical ventilation or renal replacement therapy at admission, as well as DIC and lactate level) (Additional file 2: Table S1)**.**

We looked up the number of iGAS infections requiring ICU admission in a similar pre-pandemic period (Jan 2018-May 2019) in our three centers, and found a total of 44 admitted to ICU for iGAS disease. This corroborates a higher ICU admission rate with critical GAS infections and supports the assumption of an increased incidence of critical GAS infections in the population. Also, data from the reference laboratory show an increase in invasive isolates of group A *Streptococcus* [10].

### *S. pyogenes* characteristics

**Fig. 1:**
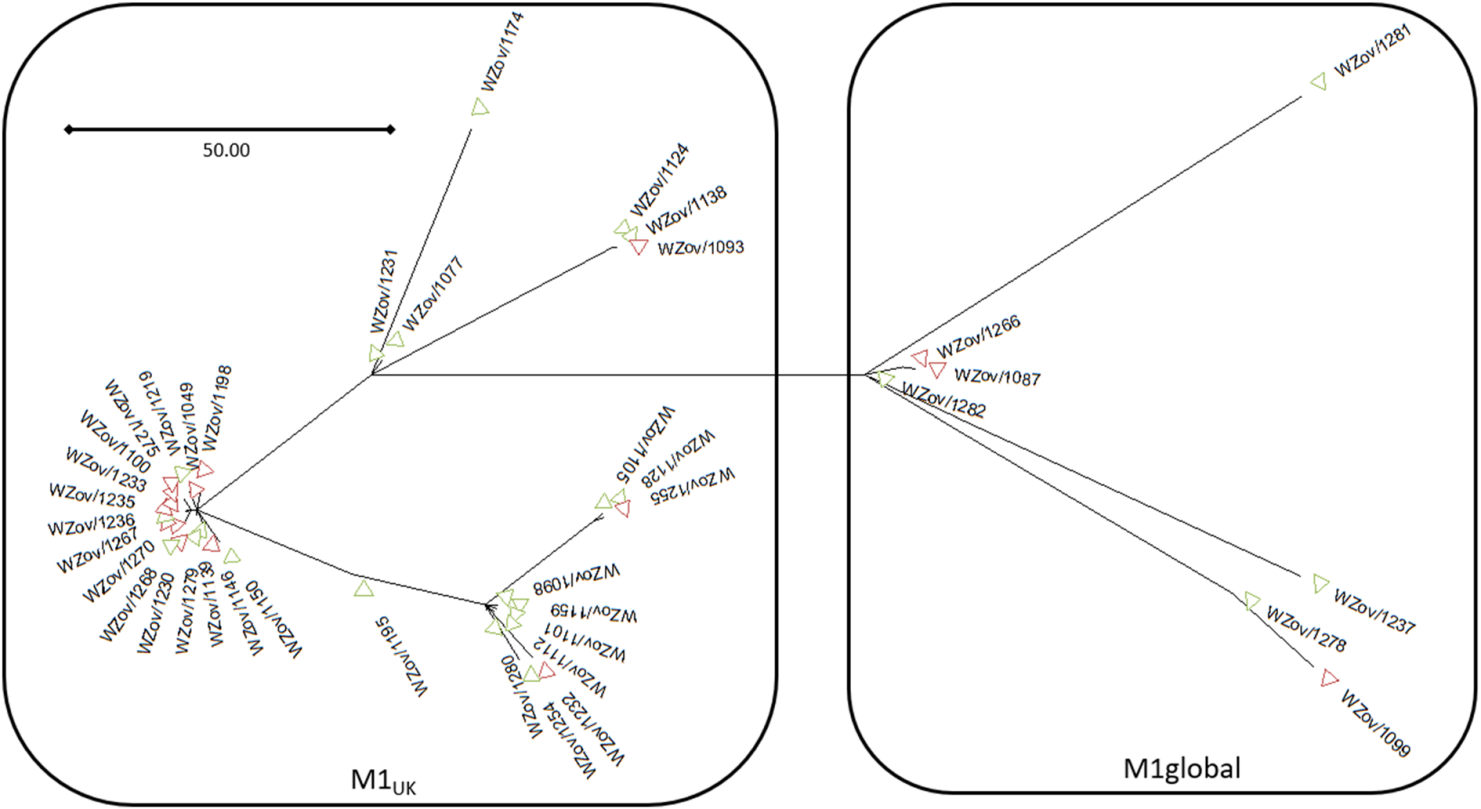
Maximum likelihood phylogenetic tree constructed from core SNPs of the 40 *emm1 S. pyogenes* isolates, with MGAS5005 as reference, differentiating between the M1_global_ and M1_UK_ lineage. Green: adult (*n* = 24), red: pediatric (*n* = 16) strains

For 55 out of the 86 patients (36/56 adult and 19/30 pediatric patients) the *S. pyogenes* strain was sent to the Belgian Reference Centre at the Antwerp University Hospital for typing. *Emm* typing showed a predominance of *emm1* (40/55, 73%) among *emm4* and *emm75* (both 2/55, 4%) and other *emm*-types occurring only once (*emm12, emm22, emm65, emm76, emm87, emm89, emm94*). Whole genome sequencing of the *emm1* strains revealed that most of them (33/40, 83%) belonged to the toxigenic M1_UK_ lineage while the M1_global_ lineage was underrepresented (7/40, 17%) (Fig. 1). Pneumonia and viral coinfection were more frequent in patients infected with *emm1* strains. We did not find significant differences in severity of illness (APACHE II), length of stay or mortality between patients infected with *emm1* *S. pyogenes* strains as compared to patients with other *emm*-types (Additional file 3: Table S2).

## Discussion

We report our recent observation of a high number of admissions to Belgian tertiary critical care units of patients with severe invasive *S. pyogenes* infections, with a striking predominance of cases with pneumonia and empyema. These patients, both adults and children, were severely ill, as reflected by the high need for mechanical ventilation, cardiovascular support and even extracorporeal membrane oxygenation. They required prolonged ICU and hospital stays. Mortality was 21% in adults and 3% in children.

This rise in critically ill patients with iGAS infections parallels the increase in hospital admissions for iGAS that has been noted in different countries worldwide in 2022–23 [11, 12, 23–26], although the clinical presentations have not been clearly described before in a large cohort, and these health alerts have mainly stressed pediatric cases.

Our data show that critical iGAS infections affect both adults and children and are associated with considerable morbidity and mortality. Case fatality rates from iGAS infections (not necessarily requiring critical care) quoted in literature are high, up to 29–45% [9], and even in young children with iGAS during 2022, 21% did not survive [23]. In adults, mortality from *S. pyogenes* pneumonia of > 20–30% has been described [27, 28]. About one in five patients with iGAS infections are admitted to ICU [29]. We found a low mortality in critically ill children and a high mortality of 21% in adults. Of note, patients with empyema and necrotizing fasciitis, as well as patients on extracorporeal support, often required multiple interventions for source control and management of bleeding complications. Finally, the length of ICU and hospital stay in our cohort also reflect the impact these severe infections have on patients and the healthcare system.

Unexpectedly, our critically ill patients had a predominance of pneumonia (56%) as clinical presentation and more than half of patients had toxic shock syndrome, which is different from previous findings of 29–39% pneumonia and 10–16% toxic shock syndrome in iGAS infections in critical care [29, 30] and from the Belgian data mentioned earlier (4% pneumonia, 10% toxic shock) [7]. Despite the fact that pneumonia was not mentioned as a common clinical presentation of iGAS infections in a recent review [31], and that *S. pyogenes* was only a rare cause (< 1%) of CAP in the CDC EPIC study 10 years ago [32, 33], we believe that intensivists and clinicians today should not discard the possibility of severe *S. pyogenes* CAP, especially after viral respiratory tract infections in the winter season [34]. It has been hypothesized that widespread pneumococcal vaccination may play a role in an increasing incidence of *S. pyogenes* CAP in children [35].

While influenza and other viral infections are often found in patients with severe or critical GAS pneumonia [28, 36, 37] and GAS incidence is higher during winter months [5], many cases of iGAS infections occur in the absence of documented viral coinfection, so other pathogen- and/or host-specific factors are likely at play.

*Streptococcus pyogenes* produces many surface-bound and extracellular virulence factors that contribute to the pathogenesis of iGAS infection [38]. The predominance (73%) of *emm1 S. pyogenes* strains in our study is striking as this type, although the most prevalent among the different *emm*-types in Belgium, usually represents around 20% of Belgian iGAS strains like in other high income countries [19]. All the *emm1* strains carried streptococcal pyrogenic exotoxins (Spe) and streptococcal mitogenic exotoxin (Sme) virulence genes *speA*, *speG*, *speJ* and *smeZ*, superantigen genes associated with invasive disease and severity of presentation [39]. Furthermore, the dominance of the toxigenic M1_UK_ variant among *emm1* isolates suggests replacement of circulating *emm1* strains in Belgium.

The M1_UK_ variant was first described in 2019 [20], has been associated with iGAS meningitis cases in the Netherlands [4], and seems to have become dominant in Belgium, as recently reported in the Netherlands and the UK [28]. This specific variant is characterized by 27 single nucleotide polymorphisms of which some have shown to increase the expression of exotoxin *SpeA* explaining its more toxigenic nature. It is associated with increase in scarlet fever and might explain the observed severity. Indeed, a recent Danish study showed that patients infected with *emm1* variants more often required intensive care treatment although mortality rate and length of hospital stay did not differ [40], in keeping with our findings. Further clinical studies are required to assess whether iGAS infections caused by the M1_UK_ variant are more severe and to discriminate the overlapping association between invasive disease, *S. pyogenes* types and clones and superantigen carriage although unraveled to some extent for specific *emm* types [41].

The relaxing of nonpharmacological infection control measures such as hand washing, face masks and social distancing could also contribute to the observed rise in iGAS [42], and potentially other invasive infections. Interestingly, the national reference laboratory for *Streptococcus pneumoniae* also saw an increase in notifications during December 2022 and January 2023, compared to the average of the years 2015–2019 [43].

Although our study describes extensively the patient and pathogen characteristics of iGAS infections requiring intensive care treatment, some limitations are of note. Since our hospitals are all tertiary care centers and the patients involved were often referred from peripheral hospitals, the characterized patient population is rather specific and might not reflect the general iGAS patient population, both in terms of disease and treatment characteristics as well as pathogen specifications. Even though the study combines data from four hospitals during a time frame of over a year, the sample size is rather limited and there is no comparison with an observation during the same time period in the pre-COVID-19 era. Both adult and pediatric iGAS infections are well represented in this study although some iGAS patients most probably were missed as *S. pyogenes* had to be isolated to be included. In case antibiotic treatment was already initiated before sample taking, this might have hampered the chances of *S. pyogenes* growth especially since molecular assays for *S. pyogenes* detection in clinical samples other than throat swabs are not routinely used [44]. Even if *S. pyogenes* was cultured, in only 64% of the cases the strains were preserved and available for further typing at the reference laboratory. Also extensive screening for viral (respiratory) co-infections is not systematically performed in clinical practice which complicates assessing their possible association with iGAS.

Faced with this new reality of severe GAS infections, a multifaceted approach seems warranted. Heightened awareness of the diverse disease manifestations and potential severe clinical course of *S. pyogenes* infections is required, as early treatment, antibiotics, source control, clindamycin and IVIG could improve outcome [2]. Indeed, there seems to be room for improvement in the use of adjunctive clindamycin and IVIG, as also seen in recent data from Australasia [45]. In our series, immunoglobulins as well as clindamycin were administered very early in the majority of the patients (certainly when presenting with necrotizing fasciitis or severe toxic shock). Moreover, beta-lactam antibiotics were started early too in the course of iGAS infection, though with a median duration somewhat longer than recommended [2] which can be explained by a high incidence of persistent abscess formation at different sites (including empyema, arthritis, spondylodiscitis or mastoiditis).

We speculate that reinforcement of face mask use in healthcare professionals [46] and in the community for people with coryzal symptoms or pharyngitis, could prevent cases of GAS disease, as it is a human host-restricted pathogen. A similar effect of nonpharmacological measures has been shown for pneumococcal disease even though it did not prevent pharyngeal carriage [42]. Also, prompt antibiotic treatment of streptococcal pharyngitis and scarlet fever could prevent household or school clusters of infection [25], and maybe even avoid invasive disease. Finally, vaccines that reduce invasive GAS infections without immunological adverse effects, have been eagerly awaited for years [47].

## Conclusions

This multicenter, retrospective study describes patient and pathogen characteristics of increased iGAS infections in Belgian tertiary care hospitals from January 2022 until May 2023. The large proportion of patients presenting with severe CAP especially in combination with a viral co-infection, mostly influenza virus is remarkable, highlighting the importance of considering GAS as a causative pathogen in severe CAP. Although the explanation for the observed epidemiological pattern of increased iGAS cases is still unclear, the introduction and replacement of the M1_global_ *S. pyogenes* clone by the toxigenic M1_UK_ strain may play a role. Reduced GAS transmission and exposure during COVID-19-related restrictions (social distancing and/or masking) and possibly an immune debt may have enhanced the rapid expansion of individual lineages. The high circulating rates of respiratory viruses during the 2022–2023 winter season, including influenza and COVID-19, may have predisposed to subsequent iGAS infection.

### Supplementary Information

**Additional file 1: Fig. S1.** Seasonal distribution of critical *S. pyogenes* infections in four Belgian centers.

**Additional file 2: Table S1.** Clinical data from 56 adult critically ill patients with invasive group A streptococcal infections, with univariate analysis of factors associated with mortality. Significant p-values are highlighted in boldface.

**Additional file 3: Table S2.** Clinical data from 55 critically ill patients with invasive group A streptococcal infections, for whom strain subtyping was performed. Comparison was made between patients infected with an *emm1 S. pyogenes* strain, as compared to patients infected with other strains.

## Data Availability

The datasets used and analyzed during the current study are available from the corresponding author on reasonable request.

## Abbreviations

APACHE II: Acute physiology, age and chronic health evaluation
BMI: Body mass index
CAP: Community acquired pneumonia
CDC: Centers for Disease Control
COVID: Coronavirus disease
DIC: Diffuse intravascular coagulation
ECMO: Extracorporeal membrane oxygenation
ENT: Ear, nose and throat
GAS: Group A *Streptococcus*/streptococcal
HMPV: Human metapneumovirus
iGAS: Invasive group A *Streptococcus*/Streptococcal
ICU: Intensive care unit
NA: Not applicable
RRT: Renal replacement therapy
RSV: Respiratory Syncytial Virus
*S. pyogenes*: *Streptococcus pyogenes*
sCAP: Severe community-acquired pneumonia
SOFA: Sequential organ failure assessment
SSTI: Skin or soft tissue infection
TSS: Toxic shock syndrome
StrepTSS: Streptococcal toxic shock syndrome
WHO: World Health Organisation
