# Supplementary material for: Clinical and molecular epidemiological features of critically ill patients with invasive group A Streptococcus infections: a Belgian multicenter case-series
Source: Ann Intensive Care. 2024 Jan 29;14:19. doi: 10.1186/s13613-024-01249-7 (PMC10825083; doi:10.1186/s13613-024-01249-7)
Supplement: Supplementary file 1 — Additional file 1: Fig. S1. Seasonal distribution of critical S. pyogenes infections in four Belgian centers. [file 13613_2024_1249_MOESM1_ESM.docx]

**Figure S1**

Seasonal distribution of critical *S. pyogenes* infections in four Belgian centres.
