# Supplementary material for: Clinical and molecular epidemiological features of critically ill patients with invasive group A Streptococcus infections: a Belgian multicenter case-series
Source: Ann Intensive Care. 2024 Jan 29;14:19. doi: 10.1186/s13613-024-01249-7 (PMC10825083; doi:10.1186/s13613-024-01249-7)
Supplement: Supplementary file 2 — Additional file 2: Table S1. Clinical data from 56 adult critically ill patients with invasive group A streptococcal infections, with univariate analysis of factors associated with mortality. Significant p-values are highlighted in boldface. [file 13613_2024_1249_MOESM2_ESM.docx]

**Table S1.** Clinical data from 56 adult critically ill patients with invasive group A streptococcal infections, with univariate analysis of factors associated with mortality. Significant p-values are highlighted in boldface.

|  | **Survivors (n=44)** | **Non-survivors (n=12)** | **p-value** |
| --- | --- | --- | --- |
| **Demographics** | | | |
| Age (years) | 46 ± 15 | 55 ± 15 | 0.0766 |
| Male gender (n, %) | 25 (57%) | 9 (75%) | 0.2530 |
| Weight (kg) | 78 ± 15 | 87 ± 28 | 0.2960 |
| BMI (kg/m^2^) | 25 ± 4 | 30 ± 8 | 0.1001 |
| **Clinical presentation** | | | |
| Duration of symptoms prior  to hospital admission (days) | 2 (1-5) | 1 (1-3) | 0.1521 |
| Pneumonia (n, %) | 21 (48%) | 4 (33%) | 0.3740 |
| with empyema (n) | 10 | 2 | 0.9304 |
| Necrotizing fasciitis (n, %) | 8 (18%) | 5 (42%) | 0.0876 |
| ENT infection (n, %) | 6 (14%) | 1 (8%) | 0.6225 |
| Puerperal sepsis (n, %) | 2 (5%) | 0 | - |
| Toxic shock syndrome (n, %) | 32 (73%) | 11 (92%) | 0.1684 |
| Viral co-infection (n, %) | 15 (34%) | 3 (25%) | 0.5500 |
| Influenza | 12 | 2 | - |
| HMPV | 2 | 1 | - |
| RSV | 1 | 0 | - |
| **Severity of illness and organ support** | | | |
| APACHE II score | 20 ± 8 | 30 ± 10 | **0.0005** |
| SOFA score at ICU admission | 11 ± 5 | 15 ± 4 | **0.0030** |
| Highest SOFA score in ICU | 12 ± 5 | 20 ± 2 | **<0.0001** |
| Invasive ventilation at ICU admission (n, %) | 24 (55%) | 11 (92%) | **0.0186** |
| Invasive ventilation (n, %) | 32 (73%) | 11 (92%) | 0.1684 |
| ventilator days | 12 (4-24) | 4 (2-29) | 0.5680 |
| Thrombocytopenia (n, %) | 17 (39%) | 7 (58%) | 0.2216 |
| Overt DIC (n, %) | 7 (16%) | 7 (58%) | **0.0026** |
| Acute liver injury (n, %) | 16 (36%) | 7 (58%) | 0.1703 |
| RRT at ICU admission (n, %) | 6 (14%) | 7 (58%) | **0.0012** |
| RRT (n, %) | 9 (20%) | 7 (58%) | **0.0100** |
| days on RRT | 15 (11-23) | 4 (2-30) | 0.4521 |
| Requiring vasoactive drugs at ICU admission (n, %) | 33 (75%) | 12 (100%) | 0.0533 |
| Lactate at ICU admission (mmol/L) | 3.9 (1.7-5.8) | 8.9 (3.0-11.8) | **0.0071** |
| Septic shock (n, %) | 37 (84%) | 12 (100%) | 0.1396 |
| Cardiogenic shock (n, %) | 14 (32%) | 6 (50%) | 0.2440 |
| ECMO (n, %) | 8 (18%) | 3 (25%) | 0.5982 |
| days on ECMO | 14 ± 5 | 27 ± 29 | 0.5243 |
| **Therapy** | | | |
| Clindamycin (n, %) | 40 (91%) | 10 (83%) | 0.4520 |
| IVIG (n, %) | 21 (48%) | 3 (25%) | 0.1585 |
| **Outcomes** | | | |
| ICU length-of-stay (days) | 16 (6-44) | 7 (2-32) | 0.0709 |
| Hospital length-of-stay (days) | 36 (22-69) | 7 (2-35) | **0.0004** |

Abbreviations: BMI = body mass index, n = number of patients, DIC = diffuse intravascular coagulation, ENT = ear, nose and throat, HMPV = human metapneumovirus, RSV = Respiratory Syncytial Virus, APACHE II = acute physiology, age, chronic health evaluation; IVIG = intravenous immunoglobulins; SOFA = sequential organ failure assessment, RRT = renal replacement therapy, ECMO = extracorporeal membrane oxygenation, ICU = intensive care unit. Thrombocytopenia (platelet count < 100000/mcL), acute renal failure (creatinine > 2 mg/dL or doubling from baseline) and acute liver injury (transaminase level or bilirubin > 2 times upper limit of normal) are defined in analogy with the CDC case definition of toxic shock syndrome.
