# Supplementary material for: Clinical and molecular epidemiological features of critically ill patients with invasive group A Streptococcus infections: a Belgian multicenter case-series
Source: Ann Intensive Care. 2024 Jan 29;14:19. doi: 10.1186/s13613-024-01249-7 (PMC10825083; doi:10.1186/s13613-024-01249-7)
Supplement: Supplementary file 3 — Additional file 3: Table S2. Clinical data from 55 critically ill patients with invasive group A streptococcal infections, for whom strain subtyping was performed. Comparison was made between patients infected with an emm1 S. pyogenes strain, as compared to patients infected with other strains. [file 13613_2024_1249_MOESM3_ESM.docx]

**Table S2.** Clinical data from 55 critically ill patients with invasive group A streptococcal infections, for whom strain subtyping was performed. Comparison was made between patients infected with an *emm1 S. pyogenes* strain, as compared to patients infected with other strains.

|  | ***emm1*-type (n=40)** | **other *emm-*types (n=15)** | **p-value** |
| --- | --- | --- | --- |
| **Demographics** | | |  |
| Age (years) | 33 (4-48) | 46 (32-64) | 0.0952 |
| Children (n,%) | 16 (40%) | 3 (20%) | 0.1648 |
| Male gender (n, %) | 26 (65%) | 7 (47%) | 0.2164 |
| Weight (kg) | 70 (17-87) | 75 (58-88) | 0.5032 |
| **Clinical presentation** | | |  |
| Duration of symptoms prior  to hospital admission (days) | 3 (2-5) | 2 (0-3) | 0.0830 |
| Pneumonia (n, %) | 26 (65%) | 5 (33%) | **0.0349** |
| with empyema (n) | 20 | 0 | **0.0006** |
| Necrotizing fasciitis (n, %) | 4 (10%) | 4 (27%) | 0.1184 |
| Other SSTI (n, %) | 4 (10%) | 4 (27%) | 0.1184 |
| ENT infection (n, %) | 4 (10%) | 5 (33%) | **0.0372** |
| Puerperal sepsis (n, %) | 0 | 1 (7%) | - |
| Toxic shock syndrome (n, %) | 30 (75%) | 11 (73%) | 0.8994 |
| Viral co-infection (n, %) | 22 (55%) | 3 (20%) | **0.0203** |
| Influenza | 12 | 0 | - |
| HMPV | 4 | 1 | - |
| **Severity of illness and organ support** | | |  |
| APACHE II score | 23 ± 9 | 20 ± 10 | 0.2002 |
| *SOFAscore at ICU admission | 14 ± 4 | 8 ± 4 | **0.0007** |
| *Highest SOFA score in ICU | 15 ± 4 | 11 ± 7 | **0.0382** |
| Invasive mechanic ventilation (n, %) | 31 (78%) | 7 (47%) | **0.0275** |
| ventilator days | 11 (4-20) | 8 (2-14) | 0.7746 |
| RRT (n, %) | 9 (23%) | 2 (13%) | 0.4491 |
| Cardiogenic shock (n, %) | 12 (30%) | 3 (20%) | 0.4583 |
| ECMO (n, %) | 9 (23%) | 0 | **0.0446** |
| **Outcomes** | | |  |
| ICU length-of-stay (days) | 15 (5-36) | 7 (3-22) | 0.1250 |
| Hospital length-of-stay (days) | 27 (16-65) | 25 (20-33) | 0.6568 |
| 28 day mortality (n, %) | 6 (15%) | 3 (20%) | 0.6553 |
| ICU mortality (n, %) | 6 (15%) | 3 (20%) | 0.6553 |
| In-hospital mortality (n, %) | 6 (15%) | 3 (20%) | 0.6553 |

Abbreviations: BMI = body mass index, n = number of patients, ENT = ear, nose and throat, SSTI = skin or soft tissue infection, HMPV = human metapneumovirus, APACHE II = acute physiology, age, chronic health evaluation; SOFA = sequential organ failure assessment, RRT = renal replacement therapy, ECMO = extracorporeal membrane oxygenation, ICU = intensive care unit. * indicates only the data from adult patients were used.
